# Epidemiology of cutaneous melanoma in Blumenau, Santa Catarina state, Brazil from 1980 to 2019^☆^

**DOI:** 10.1016/j.abd.2022.06.006

**Published:** 2023-04-28

**Authors:** Nilton Nasser, Joana Laurindo da Silva, Grazielle Corrêa

**Affiliations:** Department of Dermatology, Universidade Regional de Blumenau, Blumenau, SC, Brazil

**Keywords:** Epidemiology, Health education, Melanoma, Risk factors, Skin neoplasms

## Abstract

**Background:**

The incidence and mortality of melanoma have increased in the last years in the Caucasian population. This 40-year study of melanoma incidence in Blumenau-SC shows the impact of primary prevention on the decrease of mortality.

**Objectives:**

To classify cutaneous melanomas and evaluate their incidence in Blumenau from 1980 to 2019.

**Methodology:**

This retrospective, descriptive and cross-sectional study collected 2,336 histopathological examinations of individuals living in the city, considering sex, age, primary location, histopathological type, invasion level (Clark), and tumor thickness (Breslow). The crude coefficients of annual incidence rates were calculated using the number of melanomas and the population estimated by the Brazilian Institute of Geography and Statistics between 1980 and 2019.

**Results:**

Melanoma incidence rates reached 44.26 cases/100,000 inhabitants/year and the rates by sex reached 52.87 in men and 46.73 cases in women per 100,000 inhabitants. The most affected age group was 70 years old and over, with 421 cases in men and 301 cases/100,000 inhabitants in women. Superficial spreading melanoma occurred in 64.5% of the cases, followed by nodular melanoma in 22.8%. Early diagnoses reached 1900% with Breslow < 0.5 mm.

**Study limitations:**

This study only covers histopathological reports with definitive diagnoses of cutaneous melanoma; therefore, the data are underestimated, consequently resulting in lower rates than in reality.

**Conclusions:**

The incidence of cutaneous melanoma has increased fivefold between 1980 and 2009 and early diagnosis increased as a result of health education and primary prevention.

## Introduction

Melanoma, because of its lethality, is of great medical importance and its incidence has increased in several countries over the last 40 years.^1^, ^2^, ^3^, ^4^, ^5^ Risk factors are mainly associated with the Caucasian population submitted to ultraviolet radiation, as well as family history, presence of melanocytic nevi, and immunosuppression.^2^, ^3^, ^4^, ^5^

A total of 106,110 new cases are estimated in the United States and 8,450 cases in Brazil for 2020, 4,200 in men and 4,250 in women.^6^ Melanoma morbidity rates reach 51.7 cases per 100,000 inhabitants in Australia, considered one of the highest rates worldwide.^4^

In the southern region of Brazil, melanoma skin cancer is more frequent when compared to the other regions of the country, for both sexes.^6^, ^7^

The annual incidence rates of melanoma per 100,000 inhabitants estimated in the capitals of southern Brazil in 2019 by the National Cancer Institute (*Instituto Nacional do Câncer* [INCA]), were: 8.3 (male sex - M) and 7.39 (female sex - F) in Curitiba-PR, 6.84 (M) and 7 .94 (F) in Florianópolis-SC and 9.25 (M) and 10.12 (F) in Porto Alegre-RS, rates that are obviously underestimated in relation to Blumenau.^6^, ^7^, ^8^

In Brazil, data on the incidence coefficient of cutaneous melanoma are rare in municipalities, capitals, states, and in the country itself; they are very underestimated and require specific epidemiological studies.

The municipality of Blumenau is located in the northeast of the state of Santa Catarina, latitude 26°55′10″ south, longitude 49°03′58″, and altitude 21 meters from sea level.^9^ The Caucasian population of Blumenau, consisting mostly of German and Italian descendants with phototypes I and II according to the Fitzpatrick classification,^8^ is subject to intense radiation in the summer, with a UVB-Index between 11.5 and 13.0 according to the National Institute for Space Research (*Instituto Nacional de Pesquisas Espaciais* [INPE]) and very high according to the Environmental Protection Agency/Operational Satellites (EPA/NOOA) – United States of America, therefore, being exposed to key risk factors for melanoma.^10^

The aim of this 40-year retrospective study (1980‒2009,^11^ plus 2010‒2019) was to describe the epidemiological characteristics of cutaneous melanoma in Blumenau, presenting statistical data that can be used as reference for epidemiological studies and prevention in southern Brazil. The time length of the study allows for showing the impact of health education actions on the prevention and early treatment of cutaneous melanoma.

## Methods

This is a retrospective, descriptive and cross-sectional study that collected 2,336 cases of primary cutaneous melanoma in the three pathological anatomy laboratories in the municipality: Laboratory of Cytology, Immunopathology and Pathological Anatomy (*Laboratório de Citologia, Immunopatologia e Anatomia Pathológica* [CIPAC] - between 1980 and 2019), BML (Laboratório Beatriz Moreira Leite) and Pathology - Diagnosis in Medicine.

The data collected in the protocol by the authors included characteristics of the patients (age, sex, tumor location) and histopathological aspects of the melanoma. As inclusion and exclusion criteria in the case review, only those from the municipality of Blumenau were considered and cases of patients residing in other municipalities were excluded, aiming at the fidelity of calculations of morbidity coefficients.

The incidence rates of cutaneous melanoma per 100,000 inhabitants for Blumenau were calculated based on the annual population between 1980 and 2019 estimated by the Brazilian Institute of Geography and Statistics (*Instituto Brasileiro de Geografia e Estatística* [IBGE])^9^ and cases found annually so that they can be compared with the coefficients of other cities, states and countries. Data were entered and calculated in the Excel 2016 program, adding data from 2010 to 2019 to those published from 1980 to 2009 in the Brazilian Annals of Dermatology (*Anais Brasileiros de Dermatologia*).^10^ Chi-square, Mann-Whitney, Kruskal-Wallis (non-parametric test), and Dunn’s tests, and Spearman’s correlation were utilized. The study was approved by the Research Ethics Committee for Studies conducted in Human Beings of Universidade Regional de Blumenau according to CAAE (Certificate of Presentation of Ethical Appreciation): 42905821.2.0000.5370 and number of the Consubstantiated Opinion of the Ethics Committee: 4.580.124.

## Results

The number of cases of primary cutaneous melanoma identified between 1980 and 2019 was 2,336, 44% (n = 1078) in males and 56% (n = 1,258) in females.

Table 1 shows the annual overall incidence rates and rates by sex of primary cutaneous melanoma in the municipality of Blumenau between 1980 and 2019.^11^ The overall incidence rates of 42.2 (2011) and 44.26 (2018) cases per 100,000 inhabitants/year of primary cutaneous melanoma are highlighted, as well as the rates of 52.87 (2011) and 41.7 (2018)/100,000 inhabitants in the male sex (Table 1). The incidence rates in the female sex were 46.7/100,000 inhabitants in 2018 and 44 in 2013 (Table 1).

**Table 1:** Incidence rates per year of primary cutaneous melanoma, overall and according to gender, in Blumenau-SC, from 1980 to 2019

Year	Male	Female	Overall	Year	Male	Female	Overall
1980	5.2	3.7	**4.4**	2000	13.4	13.5	13.45
1981	5	10.9	8	2001	14.5	23.5	19
1982	11.1	8.3	9.7	2002	12	23	17.5
1983	11.8	10.3	11	2003	13.2	17.7	15.45
1984	6.9	5.5	6.2	2004	13.7	19.5	16.6
1985	13.4	15	14.2	2005	13.2	20	16.6
1986	8.7	8.4	8.5	2006	15	15.7	15.35
1987	10.6	12.2	11.4	2007	14.8	31.5	23.15
1988	7.3	6.9	6.6	2008	18	23.1	20.6
1989	15.2	12.6	13.8	2009	24.1	20.3	22.2
1990	22	17.1	18.9	2010	36.3	39.31	37.83
1991	14.6	12	12.2	2011	52.87	31.9	42.2
1992	14.4	10.9	10.2	2012	41.31	39.07	40.17
1993	22.8	17.4	15.8	2013	35.34	44.09	39.8
1994	15	11.36	9.79	2014	37.27	44.02	40.71
1995	22	18.7	14.4	2015	35.53	43.39	39.54
1996	22.6	17.9	16.8	2016	38	38.67	38.34
1997	3.4	3.3	8	2017	41	39.94	39.88
1998	23	17.2	13.7	2018	41.7	46.73	44.26
1999	21.9	17.7	18.8	2019	33.13	40.62	39.95

Source: Brazilian Institute of Geography and Statistics (*Instituto Brasileiro de Geografia e Estatística* [IBGE]), Laboratory of Cytology, Immunopathology and Pathological Anatomy (*Laboratório de Citologia, Imunopatologia e Anatomia Patológica* [CIPAC]), Pathology Diagnosis in Medicine and Beatriz Moreira Leite (BML) Pathology (years 1980 to 1990).

*Per 100,000 inhabitants.

Table 2 shows an average of the incidence rates by groups of years and, as a result, it showed significance in the period from 2000 to 2004, in which the incidence of cutaneous melanoma was significantly higher in females, with p = 0.0279. No significance was identified in the other periods.

**Table 2:** Mean incidence rates of overall cutaneous melanoma, according to period and sex, in Blumenau-SC, from 1980 to 2019

Period	Male	Female	Overall	p
	(Mean ± SD)	(Mean ± SD)	(Mean ± SD)	
1980 to 1984	8 ± 3.24	7.74 ± 3.09	7.87 ± 2.99	0.9000
1985 to 1989	11.04 ± 3.26	11.02 ± 3.3	11.03 ± 3.09	0.9925
1990 to 1994	17.76 ± 4.25	13.75 ± 3.22	15.76 ± 4.13	0.1313
1995 to 1999	18.58 ± 8.5	14.96 ± 6.54	16.77 ± 7.4	0.4720
2000 to 2004	13.36 ± 0.91	19.44 ± 4.11	16.4 ± 4.26	**0.0279**
2005 to 2009	17.02 ± 4.32	22.12 ± 5.87	19.57 ± 5.55	0.1564
2010 to 2014	40.62 ± 7.22	39.68 ± 4.98	40.15 ± 5.87	0.8166
2015 to 2019	37.87 ± 3.62	41.87 ± 3.22	39.87 ± 3.86	0.1022
1980 to 2019	20.53 ± 12.32	21.32 ± 12.81	20.93 ± 12.49	0.7790

Source: Brazilian Institute of Geography and Statistics (*Instituto Brasileiro de Geografia e Estatística* [IBGE]), Laboratory of Cytology, Immunopathology and Pathological Anatomy (*Laboratório de Citologia, Imunopatologia e Anatomia Patológica* [CIPAC]), Pathology Diagnosis in Medicine and Beatriz Moreira Leite (BML) Pathology (years 1980 to 1990).

*Per100,000/inhabitants.

p, p-value of the Mann-Whitney test (non-parametric test, compares two independent samples).

The age group with the highest numerical incidence was over 55 years old, with 57% of the cases (n = 1321), with 14.9% of the cases occurring under 39 years of age (n = 343), with 95% confidence (Table 3).

**Table 3:** Numerical and percentage distribution of cutaneous melanoma by age range and sex ‒ Blumenau-SC, from 1980 to 2019

Age range	Male	%	95%CI	Female	%	95%CI	Total	%	95%CI
0 to 39 years	178	16.6	(14.45‒18.92)	165	13.44	(11.54‒15.36)	343	14.9	(13.49‒16.41)
40 to 54 years	277	26	(23.33‒28.59)	353	28.76	(26.24‒31.3)	630	27.45	(25.64‒29.29)
55 to 64 years	253	23.7	(21.16‒26.26)	288	23.47	(21.1‒25.84)	541	23.5	(21.85‒25.32)
65 to 70 years or +	359	33.7	(30.81‒36.48)	421	34.33	(31.65‒36.97)	780	34.15	(32.06‒35.94)
TOTAL	1067	100		1227	100		2294	100	

Source: Brazilian Institute of Geography and Statistics (*Instituto Brasileiro de Geografia e Estatística* [IBGE]), Laboratory of Cytology, Immunopathology and Pathological Anatomy (*Laboratório de Citologia, Imunopatologia e Anatomia Patológica* [CIPAC]), Pathology Diagnosis in Medicine and Beatriz Moreira Leite (BML) Pathology (years 1980 to 1990).

CI, Confidence Interval (incidence estimates with 95% confidence).

The Mann-Whitney test and Spearman’s correlation were used in Table 4, to verify the increase in the incidence coefficient in each period and along the age groups. Both tests belong to non-parametric statistics. The use of such statistics is justified, considering the rejection of the null hypothesis of the normality test (Shapiro-Wilks test), that is, according to the normality test performed, the data do not show a normal distribution model.

**Table 4:** Incidence rates of melanoma by age range and gender per 100,000 inhabitants, in Blumenau-SC, in the years 1980, 2008 and 2018

Age range (in years)	Crude coefficient of incidence 1980			Crude coefficient of incidence 2000			Crude coefficient of incidence 2018		
	Male	Female	Total	Male	Female	Total	Male	Female	Total
0 to 19	0	0	0	0	0	0	0	0	0
20 to 24	0	0	0	0	8.0	4.0	0	0	0
25 to 29	0	0	0	8.6	8.3	8.5	11.4	5.9	8.7
30 to 34	0	0	0	8.3	8.2	8.3	6.3	12.6	9.5
35 to 39	47.2	22.3	34.4	17.3	8.4	12.8	21.6	6.8	13.9
40 to 44	0	0	0	10.7	0	5.2	28.8	40.8	34.9
45 to 49	0	0	0	0	12	6.3	31.2	36.6	34
50 to 54	40	0	0	32.2	16.5	25.6	50.1	71.9	61.6
55 to 59	0	0	24.2	53.7	70	63	63.8	66.9	65.5
60 to 64	0	56.2	29.7	71	58	64	74.9	185.9	135.6
65 to 69	0	0	0	150	73	105	253	128.7	182.5
70 or +	66.7	44.8	53.6	125.7	57.7	83.5	421.6	301.1	344.2

	1980	2000	2018	p
Male				
Mean ± SD	12.83 ± 23.94	33.17 ± 30.52	80.23 ± 127.62	
Median ± QD	0 ± 5^a^	20.05 ± 14.7^ab^	30 ± 28.23^b^	**0.0218**
Female				
Mean ± SD	10.28 ± 19.99	36.3 ± 38.8	71.43 ± 92.17	
Median ± QD	0 ± 2.79^b^	22.5 ± 11.06^a^	38.7 ± 39.76^a^	**0.0122**
Total				
Mean ± SD	11.83 ± 18.7	37.66 ± 35.43	74.2 ± 102.21	
Median ± QD	0 ± 12.79^a^	22.45 ± 19.29^ab^	34.45 ± 36.86^b^	**0.0289**

Source: Brazilian Institute of Geography and Statistics (*Instituto Brasileiro de Geografia e Estatística* [IBGE]), Laboratory of Cytology, Immunopathology and Pathological Anatomy (*Laboratório de Citologia, Imunopatologia e Anatomia Patológica* [CIPAC]), Pathology Diagnosis in Medicine and Beatriz Moreira Leite (BML) Pathology (years 1980 to 1990).

I – SD, Standard Deviation; QD, Quartile Deviation.

II – p, p-value of the Kruskal-Wallis test (non-parametric test). Different letters represent significant differences between years (Dunn’s test). If p < 0.05, then there are significant differences.

III – R, Spearman’s correlation: Men: R (1980 and 2008) = 22.9% and R (2008 and 2018) = 97%; Women: R (1980 and 2008) = 48.6% and R (2008 and 2018) = 80.9%; Total: R (1980 and 2008) = 57% and R (2008 and 2018) = 96.7%.

As a result of this inferential part, both in men and women, the incidence coefficient had a significant increase from 1980 to 2018. On the other hand, it showed a very strong correlation in the years 2008 and 2018, indicating a higher concentration of the number of cases in older age groups.

The incidence rate in the male sex reached 253 cases/100,000 inhabitants/year in the age group of 65 to 69 years in 2018 and 421 cases per 100,000 inhabitants in the age group ≥ 70 years. In the female sex, there were 128 cases/100,000 inhabitants/year in the age group of 65 to 69 years and 301 cases in the age group ≥ 70 years (2018) as shown in Table 4.

The most frequent histopathological types were superficial spreading melanoma with 64.5% (n = 996) between 1980 and 2019, followed by nodular melanoma with 22.8 (n = 353). Lentigo maligna melanoma reached 9.4% (n = 143) and acral lentiginous melanoma, 3.3% (n = 51; Table 5).

**Table 5:** Percentage of the incidence of cutaneous melanoma histopathological type, according to sex, in Blumenau-SC, from 1980 to 2019

Histological type	Male	%	Female	%	Total	%	p
Lentigo maligna melanoma	66	9.3	77	9.2	143	9.27	0.99800
Acral lentiginous melanoma	22	3	29	3.5	51	3.31	0.66130
Superficial spreading melanoma	427	60	**569**	**68.5**	996	64.55	**0.00050**
Nodular melanoma	**197**	**27.7**	156	18.8	353	22.88	**0.00003**
TOTAL	712	100	831	100	1543	100	

Source: Laboratory of Cytology, Immunopathology and Pathological Anatomy (*Laboratório de Citologia, Imunopatologia e Anatomia Patológica* [CIPAC]), Pathology Diagnosis in Medicine and Beatriz Moreira Leite (BML) Pathology (years 1980 to 1990).

p, p-value of the Test of 2 independent proportions.

The distribution of the histopathological types according to sex showed a predominance of 68.5% (n = 569) of the superficial spreading type in women (p = 0.00050) and 60% in men (Table 5).

Table 6 shows the numerical and percentage distribution of cutaneous melanoma according to the primary location and sex, with a predominance of 49.5% (n = 366) of primary location on the trunk in men (p = 0.0001) and 35.3% (n = 267) in women.

**Table 6:** Numerical and percentage distribution of melanoma, according to primary location and sex, in Blumenau-SC, from 1980 to 2019

Primary location	Male	%	Female	%	Total	%	p
Head	**84**	**11.37**	43	5.68	127	8.49	**0.00008**
Face	96	12.99	88	11.62	184	12.30	0.42134
Trunk	**366**	**49.53**	267	35.27	633	42.31	**0.00001**
Upper limbs	98	13.26	**191**	**25.23**	289	19.32	**0.00001**
Lower limbs	95	12.86	**168**	**22.19**	263	17.58	**0.00001**
Total	739	100.00	757	100.00	1496	100.00	

Source: Laboratory of Cytology, Immunopathology and Pathological Anatomy (*Laboratório de Citologia, Imunopatologia e Anatomia Patológica* [CIPAC]), Pathology Diagnosis in Medicine and Beatriz Moreira Leite (BML) Pathology (years 1980 to 1990).

**Figure 1:**
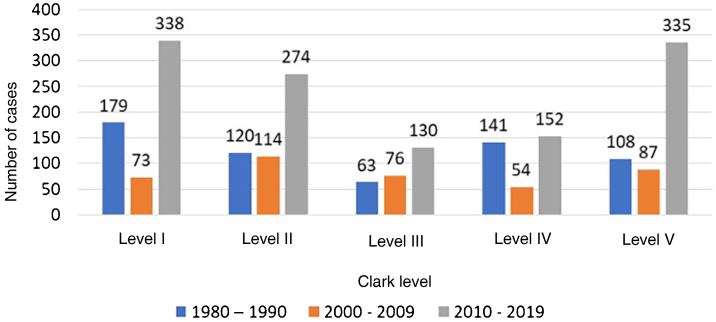
Numerical distribution of primary cutaneous melanoma, according to Clark level by decades: 1980 to 1990, 2000 to 2009 and 2010 to 2019; Blumenau-SC

Table 7 shows the percentage distribution of melanoma cases according to Clark level between 1980 and 1999, 2000 to 2009, and 2010 to 2019, highlighting the percentage increase in early diagnoses (Clark level I and II) of 24.95 % from 1980 to 1990 to 49.8% from 2010 to 2019. This means a 199.6% increase in early diagnoses from 2010 to 2019, when compared to the period 1980 to 1990.^10^ From 2010 to 2019, there was a significant increase in the number of melanoma cases in Clark levels 1 and 2 and in level 5, according to the Chi-square test. This increase can be better observed in Fig. 1.

**Table 7:** Percentage distribution of melanoma, by decades, according to the Clark level, Blumenau-SC

Clark level	1980–1990	2000‒2009	2010‒2019	p
Level I	179 (29%)	73 (18%)	338 (27.5%)	0.00001
Level II	120 (20%)	114 (27.8%)	274 (22.3%)	
Level III	63 (10%)	76 (18.70%)	130 (10.57%)	
Level IV	141 (23%)	54 (14.20%)	152 (12.36%)	
Level V	108 (18%)	87 (21.30%)	335 (27.25%)	

Source: Laboratory of Cytology, Immunopathology and Pathological Anatomy (*Laboratório de Citologia, Imunopatologia e Anatomia Patológica* [CIPAC]), Pathology Diagnosis in Medicine and Beatriz Moreira Leite (BML) Pathology (years 1980 to 1990).

p, p-value of Chi-Square Test of Independence. If p < 0.05, then there is significant association.

Table 8 shows the percentage distribution of melanomas according to Breslow’s thickness index between 1995 and 1999,^10^ 2000 and 2009,^10^ and from 2010 to 2019. Melanomas diagnosed with thickness < 1 mm reached 46.7% between 1995 and 1999^10^ and increased to 63% between 2000 and 2009,^10^ reaching 58% between 2010 and 2019 (p = 0.000001).

**Table 8:** Absolute and relative percentage frequency distribution of cutaneous melanoma, according to Breslow thickness, Blumenau-SC, from 1995 to 1999, from 2000 to 2009 and from 2010 to 2019

Breslow thickness	1995‒1999	2000‒2009	2010‒2019	p
0.0 – 0.5 mm	27 (25.23%)	140 (41.3%)	518 (42.42%)	0.000001
0.5 – 1.0 mm	23 (21.5%)	72 (21.24%)	128 (10.48%)	
1.0 – 1.5 mm	12 (11.21%)	33 (9.73%)	64 (5.24%)	
1.5 – 2.0 mm	7 (6.54%)	15 (4.42%)	35 (2.87%)	
>2.0 mm	38 (35.51%)	79 (23.3%)	476 (38.98%)	
Total	107 (100%)	339 (100%)	1221 (100%)	

Source: Laboratory of Cytology, Immunopathology and Pathological Anatomy (*Laboratório de Citologia, Imunopatologia e Anatomia Patológica* [CIPAC]), and Pathology Diagnosis in Medicine.

p, p-value of Chi-Square Test of Independence. If p < 0.05, then there is significant association.

The last period (2010‒2019) showed a significant increase in the percentage of cutaneous melanoma in the thickness categories from 0.0 to 0.5 mm and > 2.0 mm.

**Figure 2:**
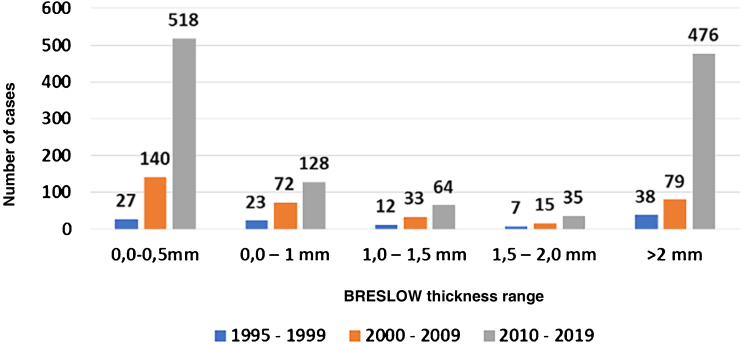
Distribution of the number of primary melanoma cases from 1995 to 1999, 2000 to 2009 and 2010 to 2019, Blumenau-SC

There was a 134% increase in the frequency of Breslow thickness between 0‒1 mm (early diagnosis) in the period 2000 and 2008^10^ when compared to the period of 1995 to 1999 and 1918% of melanoma (*in situ*) < 0.5 mm in 2010 to 2019 (518 cases), when compared to 1995–1999 (27 cases) (p = 0.000001). Fig. 2 makes this increase in the percentage number of cases clearer. Therefore, it can be said that this increase was significant according to the Chi-square test.

## Discussion

This study only considered histopathological reports with a definitive diagnosis of primary cutaneous melanoma; therefore, the data are underestimated, consequently resulting in lower rates than in reality, but high in relation to the incidence rates found in Brazil and worldwide.

The present study shows that the morbidity of cutaneous melanoma in the municipality of Blumenau increased from 4.4 (1980)^11^ to 44.26 cases per 100,000 inhabitants (Table 1), with a sharp increase in incidence, of around 1,000% in morbidity per 100,000 inhabitants compared to 1980.^11^

The world population-adjusted rates found were 25.86 melanoma cases per 100,000 inhabitants/year based on cases diagnosed between 1975‒2017 originating from 17 SEER geographic areas.^12^

As the incidence by age (SEER)^12^ was not performed and as there has been a gain in life expectancy in recent decades, part of the increase in diagnoses can be due to the extension of survival in the group of elderly people at higher risk.

The cutaneous melanoma morbidity coefficients found in Blumenau are close to those expected for European descendants (Germans and Italians), fair-skinned Caucasians living in a geographic region with a high level of ultraviolet radiation.^1^, ^3^, ^12^

### Sex

In the world population, the incidence of cutaneous melanoma in the female sex increased from 8.20^12^ in 1980 to 26.4 cases per 100,000 inhabitants (2017), with rates adjusted to the world standard. In the male sex, it increased from 9.44 in 1980^12^ to 33.31 per 100,000 inhabitants in 2017, with rates adjusted to the world standard.^12^

In England, the incidence rate adjusted to the world standard reached 24.5 cases per 100,000 inhabitants in women in 2017 and 28.8 cases in men in the same year.^13^

In Germany, with a Caucasian population such as that found in Blumenau, the morbidity coefficients found in North Rhine, Westphalia, were 13.6 cases per 100,000 inhabitants in men and 18.5 in women.^14^

Higher mortality is found in the male sex in Brazil, probably due to late diagnosis.^15^ In Blumenau, between 1980 and 2019, 2336 cases of primary cutaneous melanoma were studied, 1078 in males (46%) and 1258 in females (54%). The highest incidence rate in females reached 46.73 cases/100,000 inhabitants in 2018, and in males, 52.87 cases/100,000 inhabitants were recorded (Table 1).

There is a significant predominance of incidence in females from the year 2000 onwards due to the higher female survival in this period. The female predominance is clearly shown in Table 2. One can consider greater attention to awareness campaigns for early diagnosis and greater care for their health among women.

### Age group

Cutaneous melanoma causes more deaths than any other cutaneous tumor, and the incidence and mortality rates have increased in recent years, particularly in patients of older age groups.^15^, ^16^ From 1992 to 2006, melanoma incidence rates among non-Hispanic whites increased in all age groups. Death rates increased for older individuals (> 65 years) but not for younger people.^15^, ^16^

In the United States of America, there is a predominance of the incidence rate of cutaneous melanoma in the age group over 55 years.^17^ The incidence of melanoma in the United Kingdom and the United States of America between 1973 and 2002 increased in all age groups, both in men and women.

There was an increase from 12.4 to 56.1 cases/100,000 inhabitants in men in the age group between 55 and 64 years old, and it increased from 18.8 to 104.4/100,000 inhabitants in the United States in the group over 65 years.^17^

In this study, there was a percentage incidence of 57.65% of melanomas in the group over 54 years in the period 1980‒2019 (Table 2) and 14.9% in the age group younger than 39 years old. The crude incidence rates per 100,000 inhabitants and by age group and sex, in the years 1980, 2008 and 2018 found in Blumenau showed high morbidity rates in the age groups over 50 years old. The incidence rate in the age group of 65 to 69 years was 106 cases per 100,000 inhabitants in males in 2008 and 253 cases/100,000 inhabitants in 2018. In females in this age group of 65 to 69 years, it was 77.2 in 2008 and 128.7 cases per 100,000 inhabitants in 2018. In people aged ≥70 years, the morbidity rate per 100,000 inhabitants reached 421 cases in men and 301 cases in women in 2018 (Table 4).

Table 4 also shows the average incidence in the studied years and all the confidence tests, standard deviation, and correlation tests to make the reported data more reliable.

The high incidence of cutaneous melanoma is strongly age-related, with age-specific incidence rates rising sharply from age 50 onwards, with a peak over age 75. This high incidence indicates the need for more accurate and effective prevention programs for this age group in the municipality and in other regions with a Caucasian population similar to that of Blumenau.

### Primary location

In Canada, a 50-year study showed that the most frequent location was on the thorax (covered area) mainly in men, whereas the location on the lower limbs was more common in women, as well as in the United States in 2019.^18^

In Blumenau, the primary location of primary cutaneous melanoma was more common on the trunk, with 42.3%, with 57.8% in men, and 42.2% in women (trunk). On the lower limbs, the incidence was more common in women at 63.9% (p = 0.00001) while among men it was 36.1% (Table 6). These data can be related to the use of skirts by women and trousers by men.

### Histopathology

In a review study of 771 cases of melanoma in Texas and California, lentigo maligna melanoma was the most frequent type with 56% of cases, followed by superficial spreading melanoma with 29%.^19^, ^20^

In Blumenau, between 1980 and 2019, superficial spreading melanoma was the most frequent type, with 64.5%, mainly in females with 68.5% (p = 0.00050) and 60% in males. The second most common histopathological type was nodular melanoma with 22.8%, followed by lentigo maligna melanoma at 9.4%, and acral lentiginous melanoma at 3.3% (Table 4).

Nodular melanoma appears in all studies with higher mortality due to the more advanced level of invasion.^19^, ^20^, ^21^ Diagnostic staging was not possible, because the study was based only on histopathological reports.

### Level of invasion

Patient survival depends on the thickness and level of invasion of the primary cutaneous melanoma, and its decrease found on histopathological reports is equivalent to early diagnosis and improved survival.^21^, ^22^

Patients with primary cutaneous melanoma with a Breslow thickness < 1 mm are considered to be at low risk and have an excellent prognosis for survival, potentially leading to zero mortality.^21^, ^22^

In Blumenau between 1980 and 1990,^11^ 25% of those diagnosed with primary cutaneous melanoma showed Clark levels I and II, and between 2010‒2019 this percentage increased to 49.8% of cases (Table 7; p = 0.00001), an increase of 199%, with possible survival improvement.^10^, ^21^, ^22^ Fig. 1 clearly shows the number of early diagnoses represented by Clark levels I and II.

From 2000 to 2009,^11^ considering Breslow thickness, the percentage of early diagnosis was 62.5% for melanomas < 1 mm, whereas it was 52.85% from 2010 to 2019 (Table 8). A similar value was reported in the US, where 66% of all melanomas diagnosed between 1988 and 1999 had Breslow thickness < 1 mm.^22^, ^23^, ^24^

The present study shows, therefore, that there was an increase in early diagnoses comparing data of Breslow indexes in the period from 2000 to 2009^10^ and 2010 to 2019, with data related to the period from 1995 to 1999^11^ (Table 8).

When analyzing Table 7, Table 8, it can be observed that thin melanomas (< 1 mm Breslow and Clark levels I and II) may indicate a longer survival of patients with melanoma, which is inversely proportional to tumor thickness.^21^, ^22^ Considering “thin” melanomas, there is an increase in the possible survival improvement of 199%, according to the Clark level (Table 7) in 2019 compared to 1980,^11^ and 113% of survival according to Breslow thickness in the decade of 2010 to 2019 compared to 1995 to 1999^10^ (Table 8).

*In situ* melanoma diagnoses (0‒0.5 mm) increased by around 1,900% from 2010 to 2019 (518 cases) compared to 1995 to 1999 (27 cases); p = 0.000001). These data are best seen in Fig. 2.

The increase in invasive melanomas (> 2 mm) may be due to the increase in population survival in the municipality.

The fatality rate represented by “thick” melanomas may have decreased by 65% when comparing the percentages between 1980 to 1990^11^ and the decade 2010 to 2019.

The decrease in cutaneous melanoma thickness in histopathological reports can be attributed to population education campaigns for prevention and training of health professionals for early diagnosis, besides treatment and the use of dermoscopy by dermatologists. This evidence is demonstrated and supported in this 40-year study, which controlled for and compared levels of invasion and morbidity coefficients.^21^, ^22^, ^23^, ^24^, ^25^

## Conclusions

The results found in this study can be used as reference for most municipalities in southern Brazil where there is intense solar radiation affecting the light-skinned population, with phototypes I and II, of European descent.

The increase in early diagnosis can be attributed to education and primary prevention campaigns, as reported in other countries,^22^, ^23^, ^24^, ^25^ and carried out in Brazil by the Brazilian Society of Dermatology (*Sociedade Brasileira de Dermatologia*).

This 40-year epidemiological study concludes with the following observations:

From 1980 to 2019, the crude rate of melanoma increased from 4.4 cases^11^ to 44.26/100,000 inhabitants, with a peak of 46.73 in women and 52.87 in men and a predominance of melanoma incidence in the female sex, with 56% of cases (n = 1,258).

There was a higher incidence of melanoma in the age group over 50 (64.9%), with a peak ≥70 years, with 421.6 cases per 100,000 male inhabitants and 301 cases in females (2018). There was a predominance of the superficial spreading type, with 64.5% of cases (n = 996) followed by nodular melanoma with 22.8% (n = 353).

There was an increase in early diagnosis in the period 2000 to 2009,^10^ represented by 63% of diagnoses with Breslow thickness < 1 mm and 53% from 2010 to 2019.

A possible increase in survival^21^, ^22^, ^23^, ^24^, ^25^ of 199% according to Clark level is also verified in 2010 to 2019, compared to 1980 to 1990^11^ and 113% of survival according to Breslow thickness in the 2010 to 2019 decade when compared to 1995 to 1999.^10^

The fatality rate represented by thick melanomas decreased from 75% of cases from 1980 to 1990^10^ to 49% in the 2010 to 2019 decade.^19^, ^20^

Considering the increased incidence, especially in the elderly, it is essential to maintain public awareness campaigns about the initial signs of melanoma, the ABCDE rule, care for family members, multiple nevi, and priority care for the elderly.

## Financial support

None declared.

## Authors’ contribution

Nilton Nasser: Design and planning of the study; data collection; writing of the manuscript and critical review of the content; preparation of tables, from 1980 to 2019; approval of the final version of the manuscript.

Joana Laurindo da Silva: Data collection, legal aspects of the study, such as approval by the Ethics Committee, literature search; help preparing the tables; approval of the final version of the manuscript.

Grazielle Corrêa: Data collection, data survey; help preparing the tables; approval of the final version of the manuscript.

## Conflicts of interest

None declared.
